# Supplementary material for: Structural Analysis and Anti-Inflammatory Effect of a Digalactosyldiacylglycerol-Monoestolide, a Characteristic Glycolipid in Oats
Source: Nutrients. 2022 Oct 6;14(19):4153. doi: 10.3390/nu14194153 (PMC9570764; doi:10.3390/nu14194153)
Supplement: Supplementary file 1 [file nutrients-14-04153-s001.zip › nutrients-1931766-supplementary.pdf]

Supplementary Information 1

The data of NMR analyses of compound X

A: ^{1}H NMR (600 MHz, CD_3OD)

B: ^{13}C NMR (600 MHz, CD_3OD)

C: COSY (CD_3OD)

D: TOCSY (CD_3OD)

E: HSQC (CD_3OD)

F: HMBC (CD_3OD)

G: NOESY (CD_3OD)

H: ROESY (CD_3OD)

A: ^{1}H NMR (600 MHz, CD_3OD)

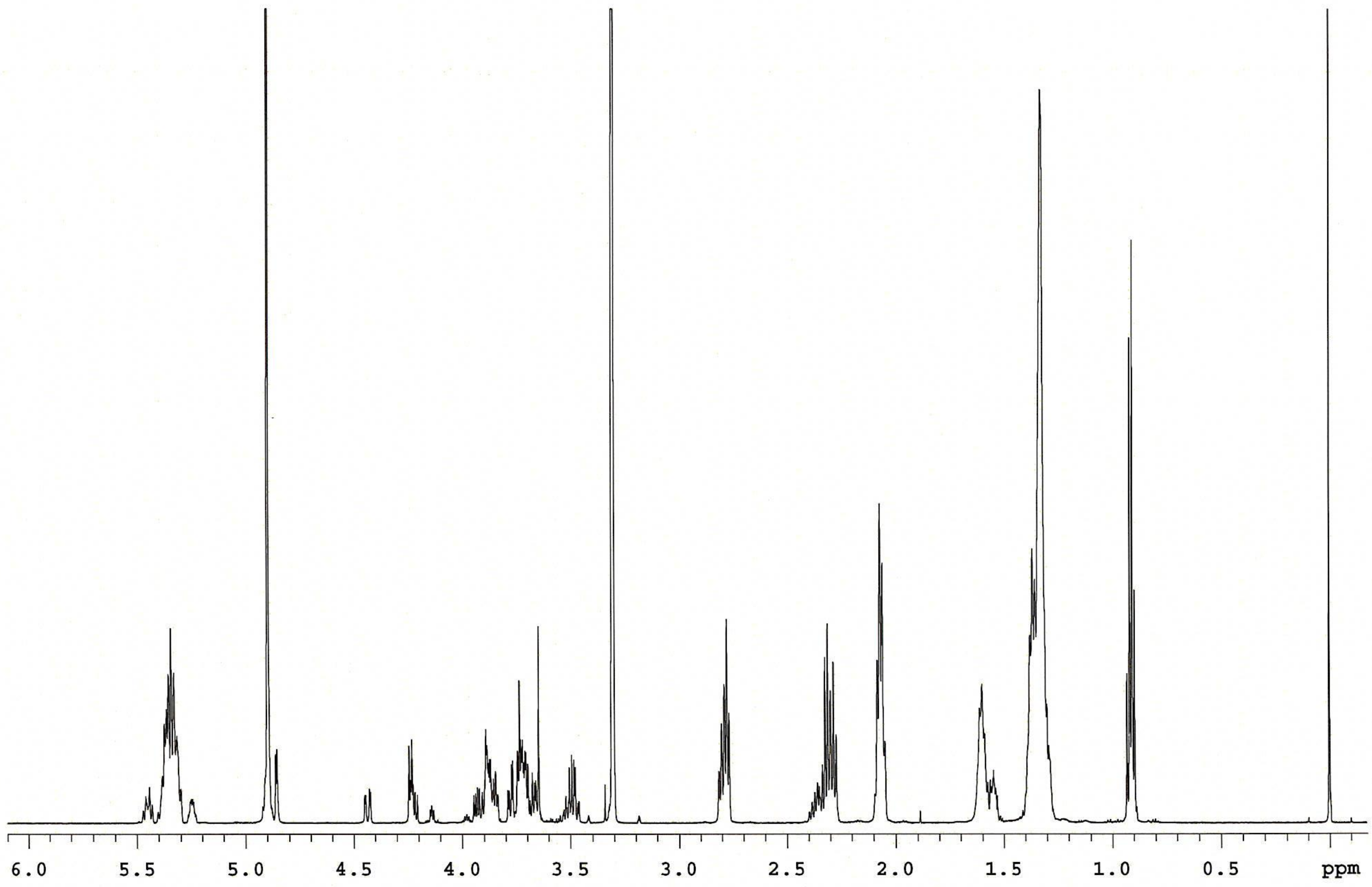

B: ^{13}C NMR (600 MHz, CD_3OD)

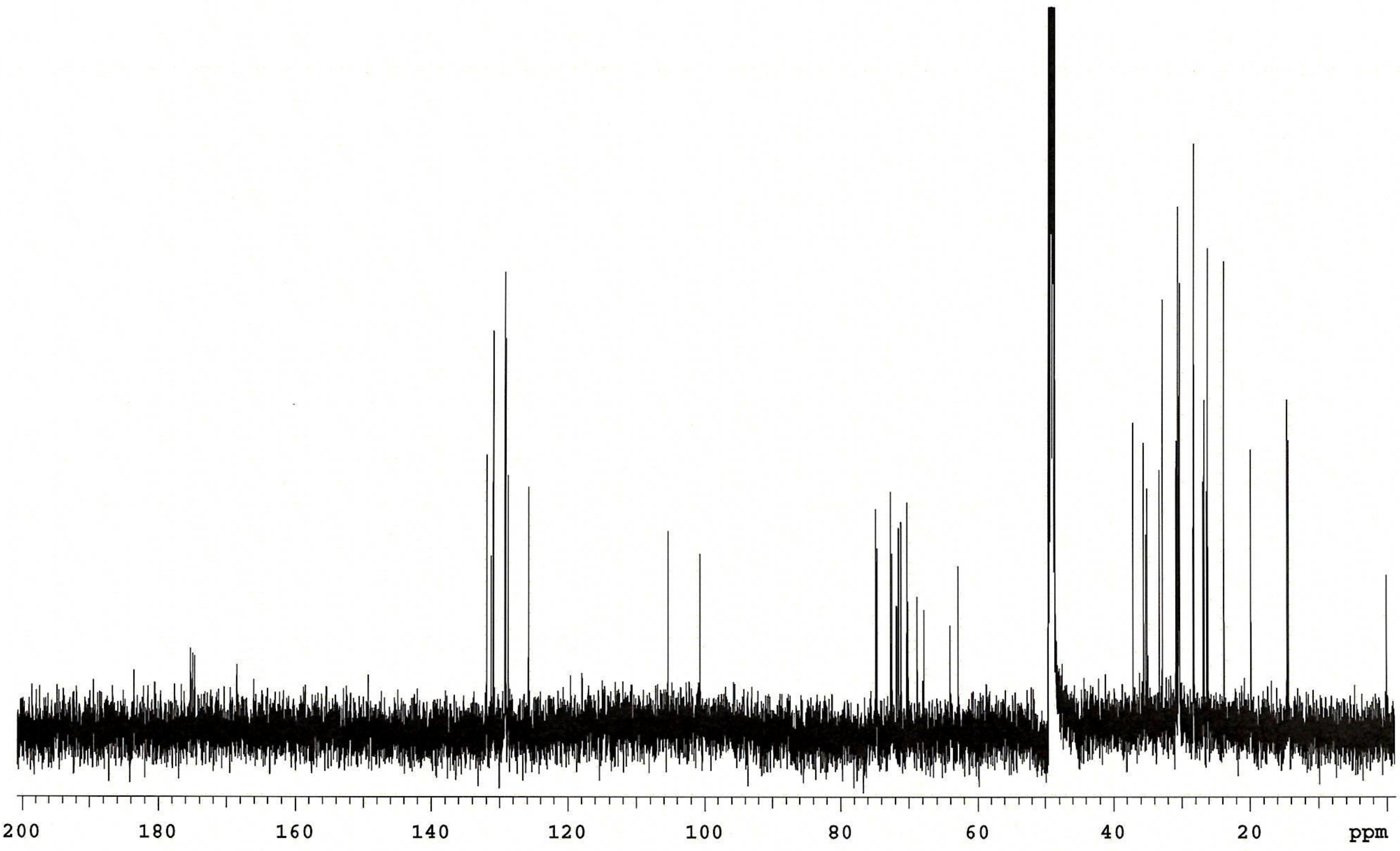

D: TOCSY (CD_3OD)

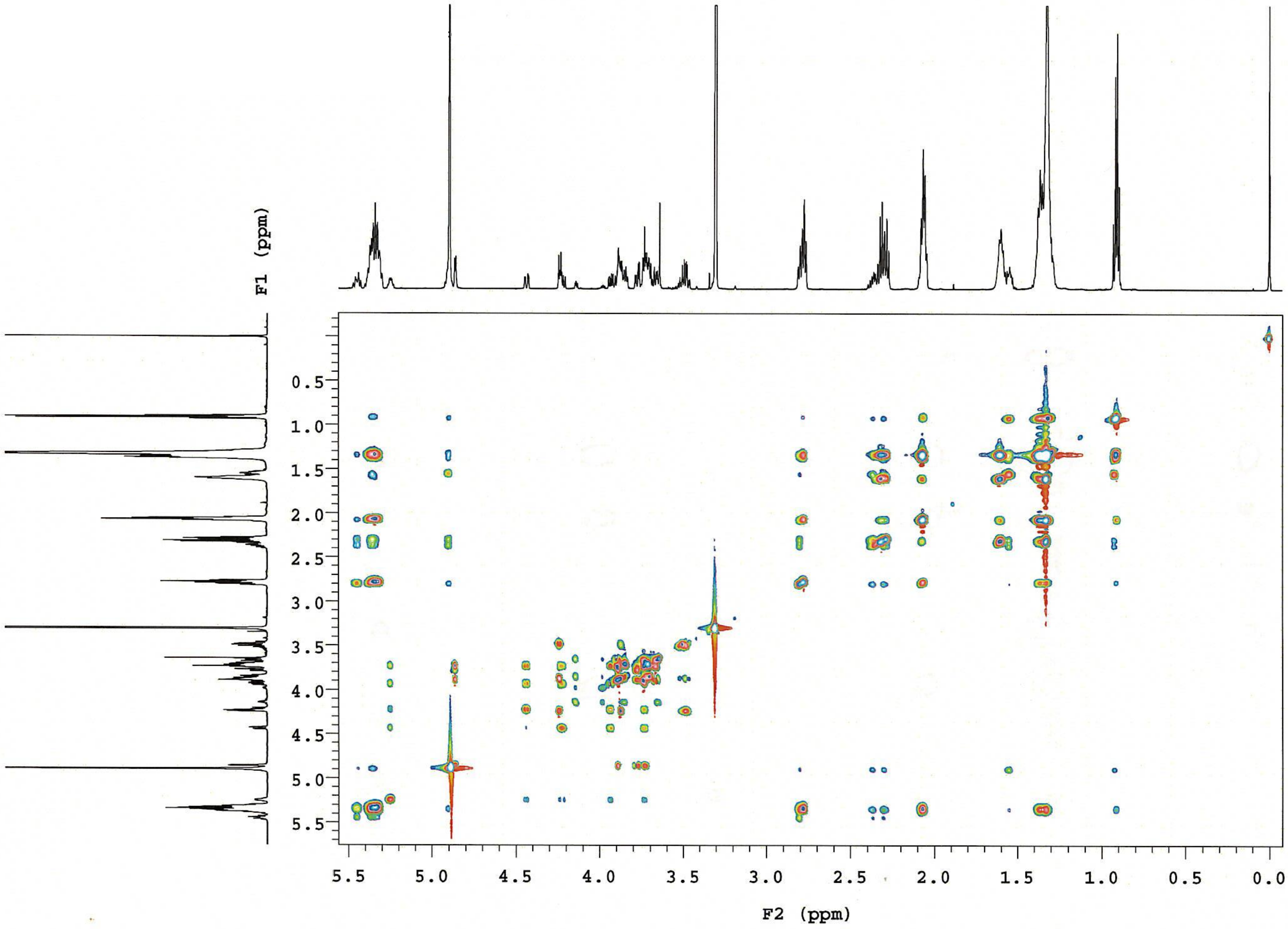

C: COSY (CD_3OD)

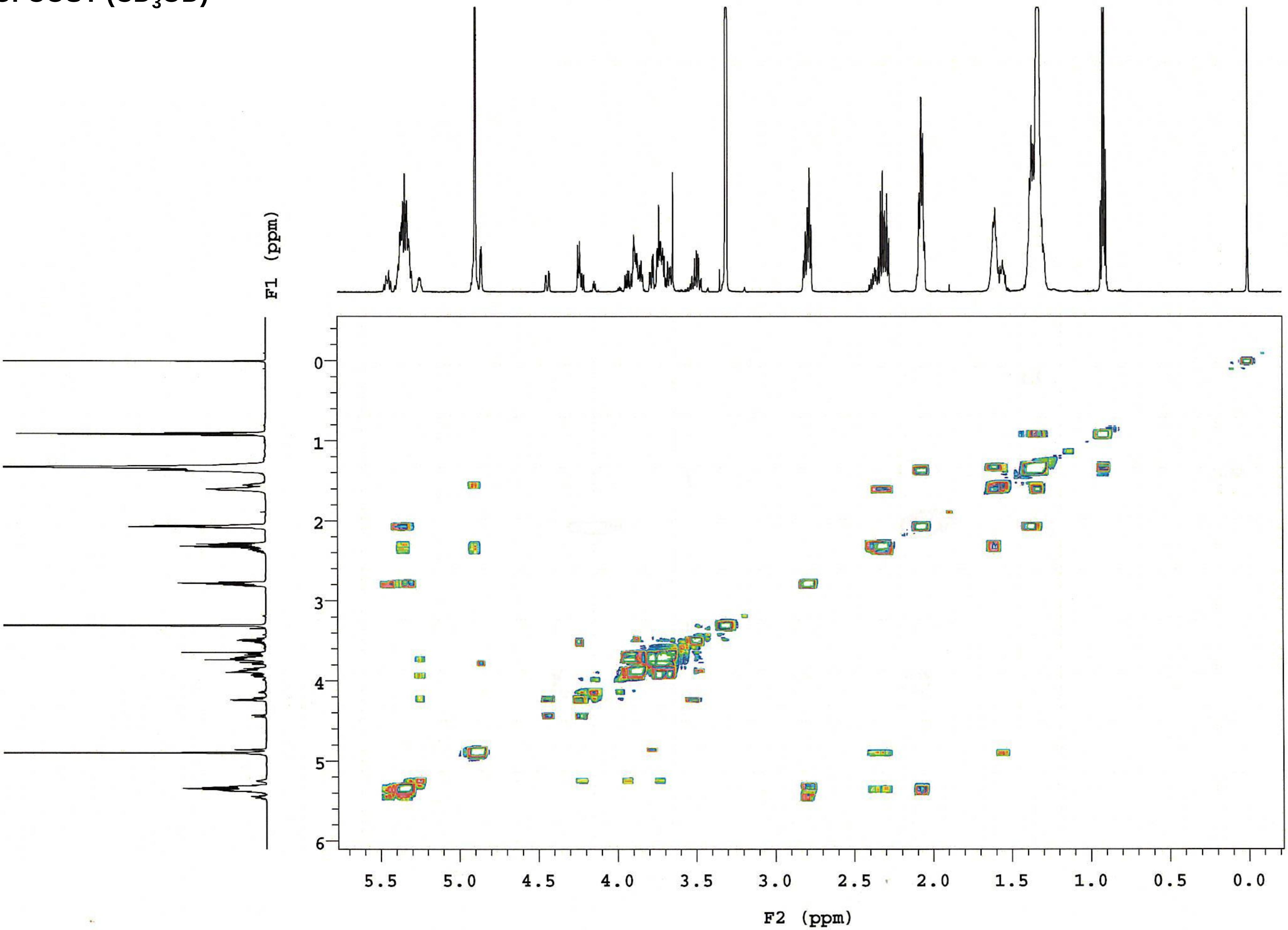

E: HSQC (CD_3OD)

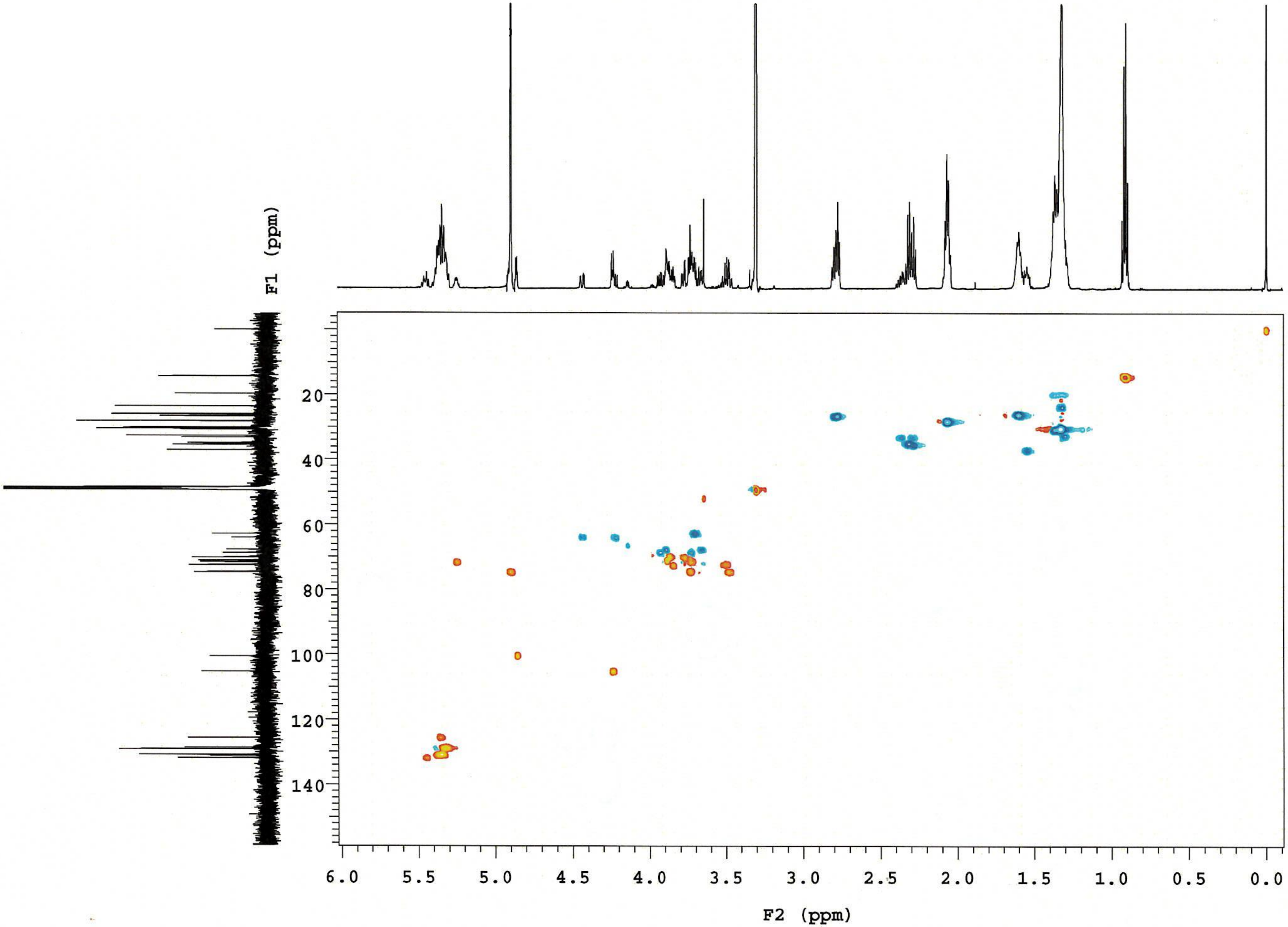
F1 (ppm)
20
40
60
80
100
120
140
6.0
5.5
5.0
4.5
4.0
3.5
3.0
2.5
2.0
1.5
1.0
0.5
0.0
F2 (ppm)

F: HMBC (CD_3OD)

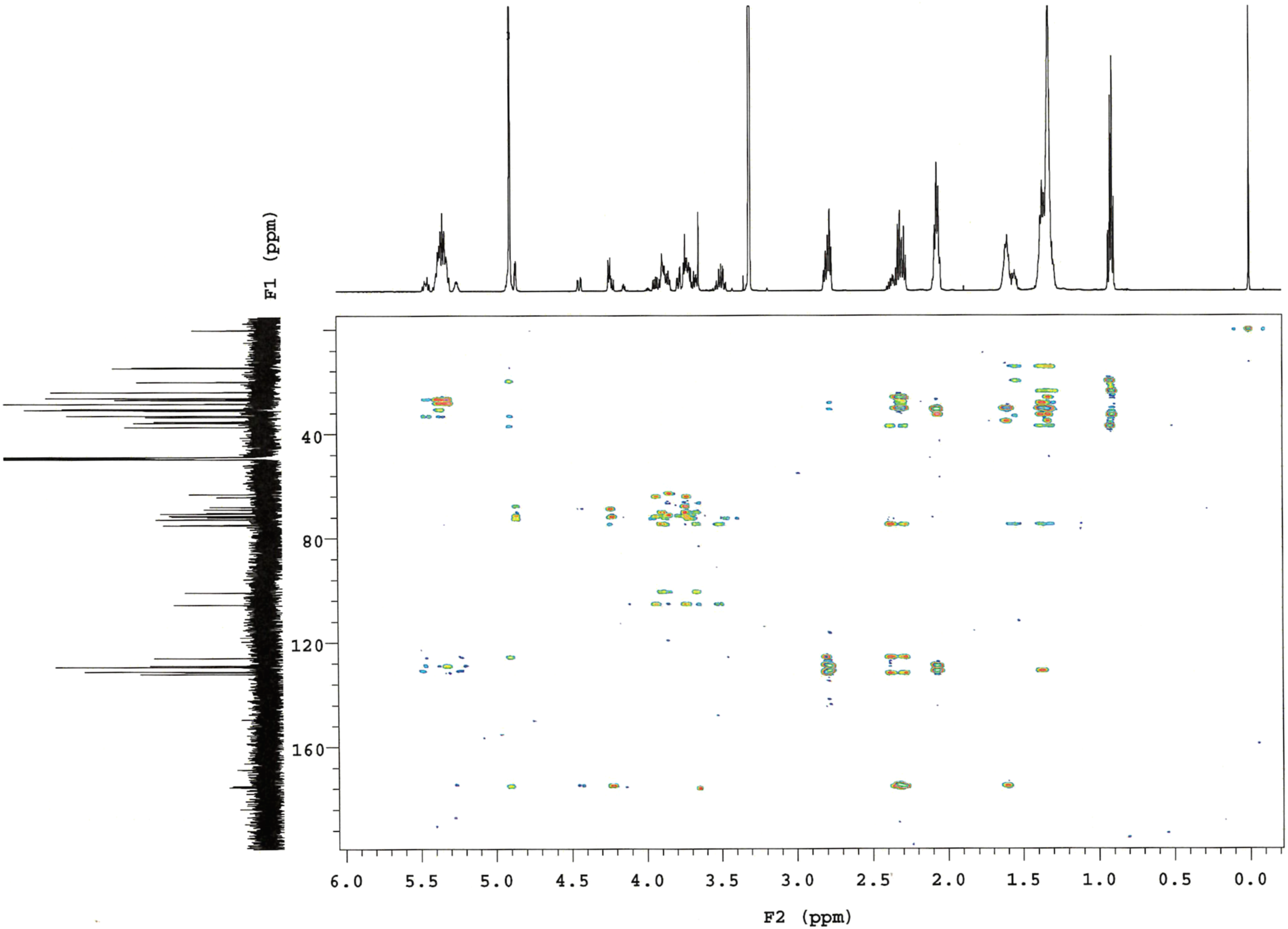

G: NOESY (CD_3OD)

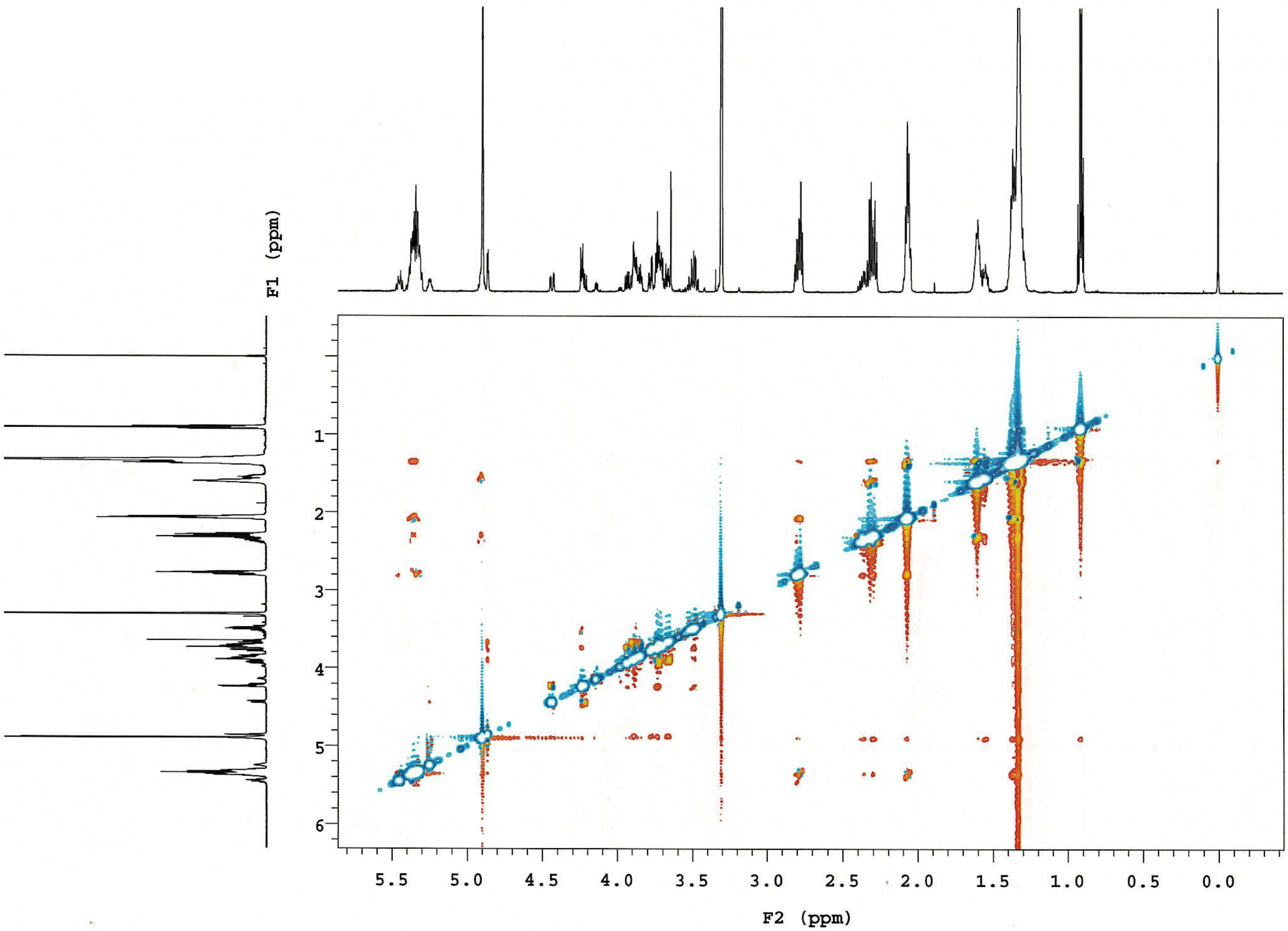

H: ROESY (CD_3OD)

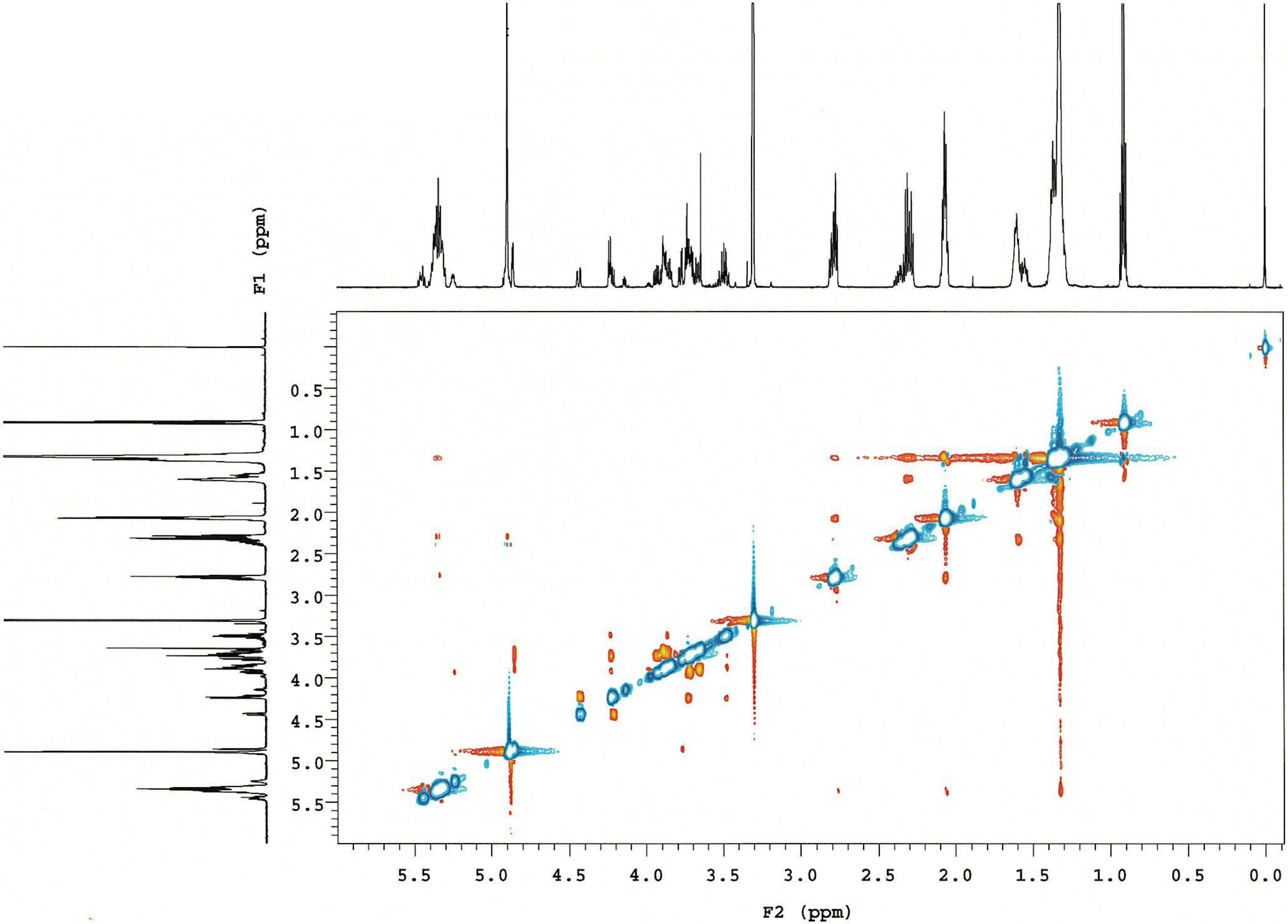

Supplementary Information 2

LC-MS/MS analysis of compounds that could arise from the DGDG-LAHLA metabolism.

Desorption of fatty acids (LA or LAHLA)

**Desorption of galactose (Gla)**	**None**	**LA (*sn*-1)**	**LA (LAHLA)**	**LA (*sn*-1), LA (LAHLA)**	**LAHLA (*sn*-2)**
**None**	DGDG-LAHLA<br>*m/z* 1241.6 > 961.5	A<br>*m/z* 979.6 > 699.6	B<br>*m/z* 979.6 > 683.6	C<br>*m/z* 717.4 > 555.4	D<br>*m/z* 701.4 > 539.4
**Gla**	E<br>*m/z* 1079.8 > 799.8	F<br>*m/z* 817.5 > 537.5	G<br>*m/z* 817.5 > 521.5	H<br>*m/z* 555.3 > 259.3	I<br>*m/z* 539.3 > 259.3
**Gla, Gla**	J<br>*m/z* 917.7 > 637.7	K<br>*m/z* 655.5 > 375.5	L<br>*m/z* 655.5 > 359.5	M<br>*m/z* 393.3 > 97.3	N<br>*m/z* 377.3 > 97.3

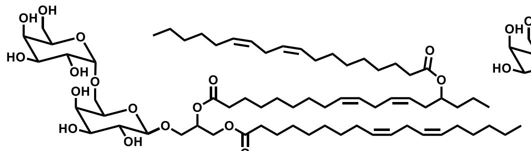

DGDG-LAHLA

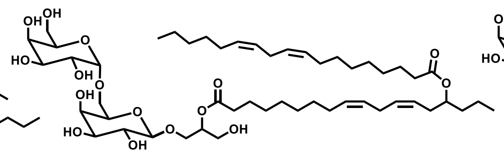

A

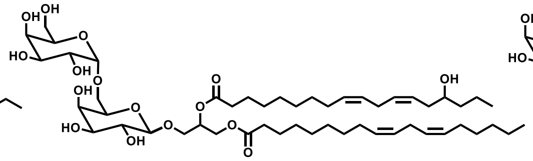

B

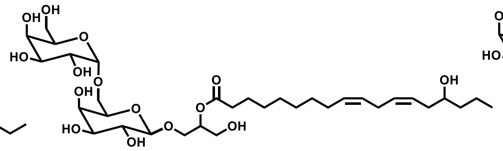

C

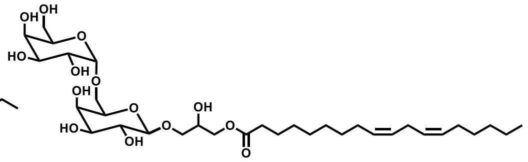

D

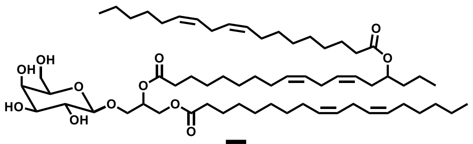

E

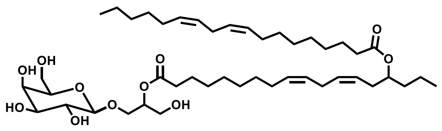

F

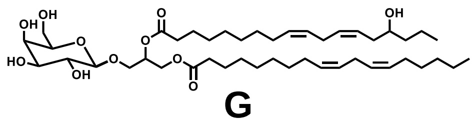

G

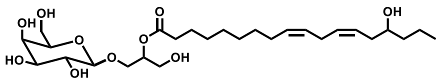

H

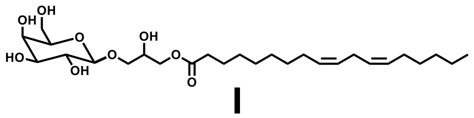

I

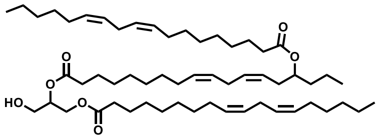

J

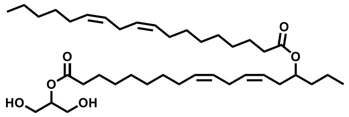

K

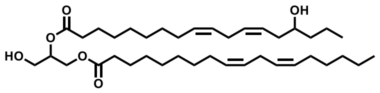

L

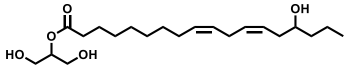

M

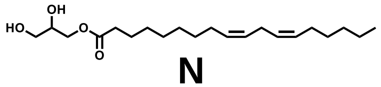

N

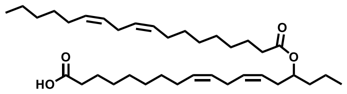

15-LAHLA

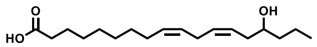

15-HLA

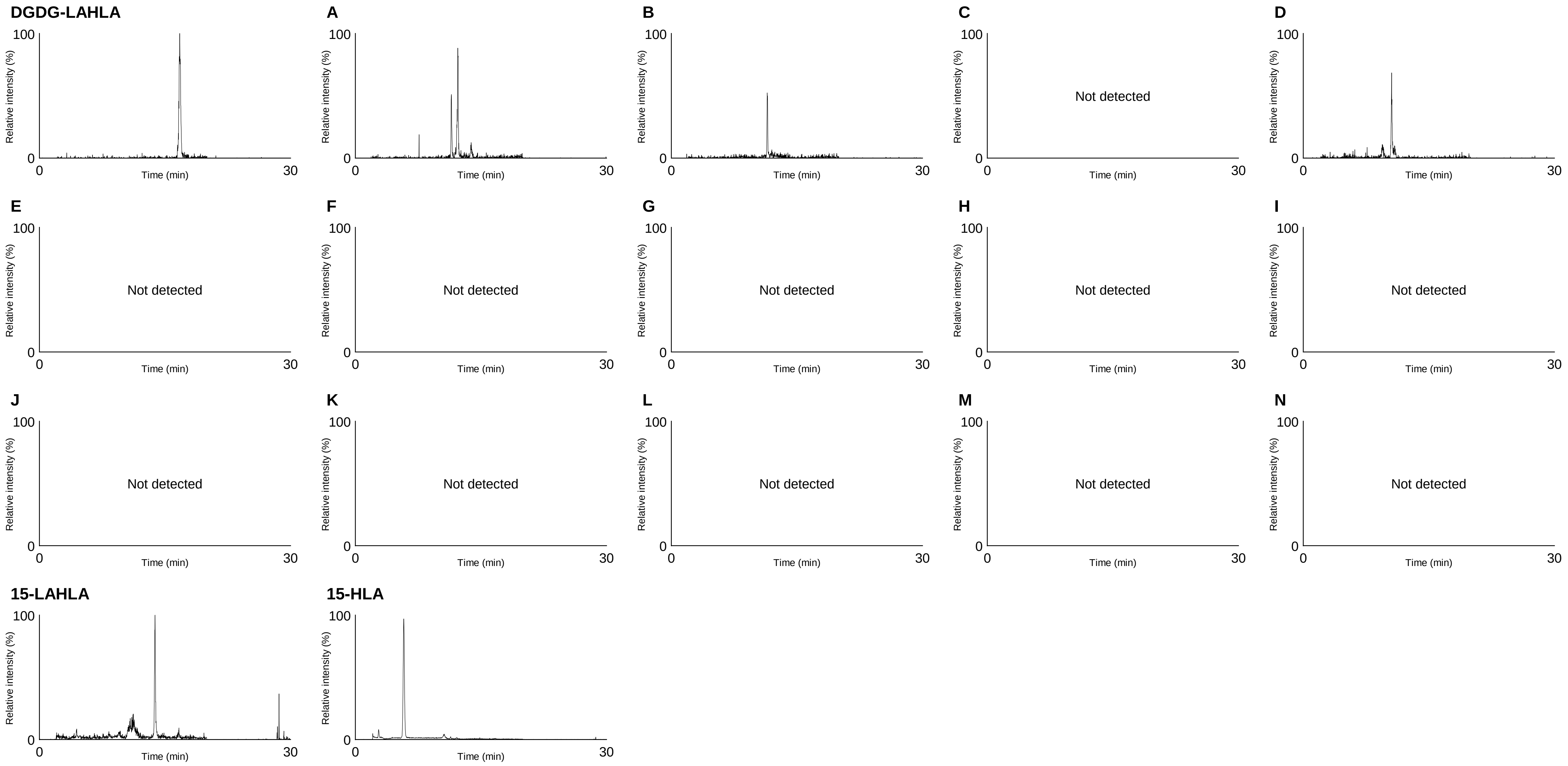
DGDG-LAHLA
A
B
C
D
E
F
G
H
I
J
K
L
M
N
15-LAHLA
15-HLA
Relative intensity (%)
Time (min)
100
0
30
Not detected
